# Investigating the role of self-construal in the formation of entrepreneurial intentions

**DOI:** 10.3389/fpsyg.2015.01085

**Published:** 2015-07-29

**Authors:** Leonidas A. Zampetakis, Konstantinos Kafetsios, Manolis Lerakis, Vassilis Moustakis

**Affiliations:** ^1^Management Systems Laboratory, School of Production Engineering and Management, Technical University of CreteChania, Greece; ^2^Department of Psychology, School of Social Sciences, University of CreteRethymnon, Greece

**Keywords:** independence, interdependence, entrepreneurial intentions, planned behavior, self-construal, Greek students

## Abstract

Individuals may perceive themselves as independent and distinct from others or as interdependent and connected to others. Do these differences in self-construal influence entrepreneurial preferences and intentions to start a new business in university students? Few studies have examined the influence of cultural orientations on entrepreneurial intentions at the individual level of analysis. Two studies investigated the role of independent and interdependent self-construal within the theory of planned behavior (TPB). In the first study, results from structural equation modeling analyses found that chronic independent self-construal was related to attitudes toward entrepreneurship and moderated relationships between attitudes and entrepreneurial intentions. In the second study, participants primed with an independent self-construal had more favorable entrepreneurial attitudes, but not intentions than participants primed with an interdependent focus. This set of studies extends cognitive models of entrepreneurship by demonstrating the role of self-construal in the TPB model at individual level.

## Introduction

The creation of new ventures is a conscious and deliberate decision that involves considerable planning and a high degree of cognitive processing (Bird, 1988; Krueger et al., 2000). Thus, an entrepreneurial career decision can be considered the type of planned behavior for which entrepreneurial cognition models are ideally suited (Bird, 1988). Entrepreneurial intentions, in turn, are a deciding factor for performing entrepreneurial behavior. Entrepreneurship is linked to value creation and as such it is thought to have a significant impact on economic growth, business renewal, and employment. Given the significant socio-economic returns of entrepreneurship, it is important to identify the factors that influence entrepreneurial intentions in order to gain a better understanding about the drivers and inhibitors of entrepreneurship.

Cognitive models of entrepreneurship underline the importance of entrepreneurial intentions such as the engagement in business formation, as antecedents to planned behavior (Krueger et al., 2000; Peterman and Kennedy, 2003). Entrepreneurial intentions are defined as individuals' convictions to start a new venture while consciously planning to open a business at some future point of time (Bird, 1988). Among several cognitive models of entrepreneurial intentions (e.g., the entrepreneurial event model, Shapero and Sokol, 1982; entrepreneurial schema, Busenitz and Lau, 1996; the entrepreneurial potential model, Krueger et al., 2000) Ajzen's TPB, has been one of the most influential theory-driven models to explain entrepreneurial intentions. Prior applications of the TPB in the entrepreneurship literature suggest that the model's components typically explain a large part of the variance in intentions (ranging from 45 to 60%, Krueger et al., 2000; Carsrud and Brännback, 2011). According to the TPB framework, persons are motivated toward a particular behavior to the extent that they hold a positive attitude toward that behavior, a perceived social pressure to perform that behavior, which is taken to represent subjective norm, and perceive that they should be able to have control when they implement the specific behavior.

The TPB has been successfully applied to describe entrepreneurial intentions in different countries (USA, Krueger et al., 2000; Norway, Kolvereid, 1996; Spain and Taiwan, Liñán and Chen, 2009; Scandinavian countries, Autio et al., 2001; South Africa, Gird and Bagraim, 2008; Russia, Tkachev and Kolvereid, 1999). Although, arguably, the TPB model generalizes across cultures, cultural values are set to moderate the strength of the TPB components' relationships with entrepreneurial intention (e.g., Busenitz and Lau, 1996; Mitchell et al., 2000; Mueller and Thomas, 2001; Liñán and Chen, 2009). With few exceptions (Siu and Lo, 2013) this body of research has used country as a proxy variable for differences in cultural orientations' effects on entrepreneurial intentions. Yet, applying country level (mean) scores of cultural values, such as individualism and hierarchy distance, has been recognized as a significant limitation operationally (Schaffer and Riordan, 2003) and conceptually (Hofstede, 2001; Oyserman et al., 2002a).

The present studies examined how the two dimensions of a seminal cultural orientation (independent and interdependent self-construal) at individual level, relate to the TPB components and how they impact on the relative effects attitudes, subjective norms and perceived behavioral control have for entrepreneurial intentions within a given culture (Greece). Study 1 examined the role of chronic independent and interdependent self-construal on the TPB and entrepreneurial intentions. In study 2 results from study 1 were replicated with an experimental design of how temporarily manipulated self-construal may influence entrepreneurial attitudes and intentions. The present studies extend cognitive models of entrepreneurship by identifying the conditions and implications of key cultural dimensions, independence and interdependence in this case, within a single culture for judgment (Aaker and Maheswaran, 1997; Shavitt et al., 2006) and entrepreneurial preference (Bird, 1988; Busenitz and Lau, 1996).

### The role of self-construal in enterpreneurial intentions

Self-construal is a key psychological construct that concerns the distinct ways persons understand themselves in relation to their social context. It refers to individuals' culturally-contingent thoughts, feelings and actions that are concerned with one's understanding of the self as connected to others, especially to members of in-groups (interdependence) or distinct from others (independence, Markus and Kitayama, 1991; Cross et al., 2011). Independent self-construal places an emphasis on individual needs, agency, autonomy, and self-fulfillment whereas interdependent self-construal emphasizes relationships with others, obligations, and especially obligations to in-group members (Singelis, 1994; Triandis, 1995; Oyserman et al., 2002a). Hofstede's (2001) psychometric approach has been widely applied to understand cultural differences including culture-mean differences in individualism and collectivism orientations that have taken to correspond to individual-level independent and interdependent self-construal.

By involving both individual and social-contingent aspects of the cultural self, self-construal is ideally suited to explain TPB components. Some evidence on the effects of independent and interdependent self-construal in entrepreneurial cognitions comes from studies that examined the TPB antecedents to entrepreneurial intentions in different cultures. While it is likely that higher levels of the three TPB components (attitudes ATT, subjective norms SN, and perceived behavioral control PBC) may positively influence entrepreneurial intentions, it is also likely that individuals may react differently depending on their cultural orientations, independent and interdependent self-construal in particular. In individualistic societies attitudes toward entrepreneurship are typically found to be significant predictors of entrepreneurial intentions (Kolvereid, 1996; Krueger et al., 2000; Autio et al., 2001) whereas, participants' subjective norms are not predictive of entrepreneurial intent. These results are consistent with research suggesting that people with independent self-construal weight perceived attitudes more than subjective norms in behavioral decisions (e.g., Ybarra and Trafimow, 1988). In more collectivistic societies results have been mixed. A cross-cultural study across Taiwan and Spain, two cultures that differ on levels of individualism (higher in Spain), found that attitudes were significant predictors of enterpreneurial intentions in both (Liñán and Chen, 2009). However, SN had a stronger effect on entrepreneurial intentions through attitudes and perceived behavioral control in Taiwan (the more collectivistic culture) than Spain although the Taiwanese participants reported lower SN^1^ than participants in the Spanish sample. Another study also in a collectivistic context (Siu and Lo, 2013) partly confirmed the results of Liñán and Chen (2009) regarding the importance of SN but differed with regards to the predictive strength of ATT on entrepreneurial intentions: subjective norms (SN) and perceived behavioral control (PBC) were significant predictors of intentions to start a business. However, contrary to Liñán and Chen's findings, attitudes toward entrepreneurship (ATT) were a non-significant predictor of entrepreneurial intentions.

With few exceptions (i.e., Siu and Lo, 2013) this body of research has considered culture as the primary unit of analysis. While the influence of culture on entrepreneurial intentions is widely acknowledged, little research has examined the influence of cultural orientations on entrepreneurial intentions at the individual level of analysis. Generalizing relationships observed at the cultural level to that of the individual has been recognized as an ecological fallacy (Hofstede, 1991, 2001; Oyserman et al., 2002a).

When culture is the unit of analysis significant within-culture variation is ignored. Importantly, it is incorrect to assume that a culture's mean level cultural value dimensions, individualism, and collectivism in this instance, readily reflect individual-level cultural orientations, independence and interdependence, respectively (Triandis, 1995). Rather, individuals tend to sample from individualistic and collectivistic tendencies, very much depending on particular circumstances (Miyamoto, 2013). The chronic accessibility or activation of independent or interdependent mind-sets within any given culture leads to the formation of chronic, readily accessible knowledge that is equivalent to a trait (Oyserman and Sorensen, 2009). As such, self-construal can temporarily vary on the independence and interdependence dimensions when primed with appropriate situational primes such as relationships, groups, or obligations in the case of interdependence or individual traits and values in the case of independence (Gardner et al., 1999). Temporarily activated self-construal can consequently influence related cognitions (Oyserman, 2011; Miyamoto, 2013). The present research extends this rationale to examining relationships between within-culture variations in self-construal and entrepreneurial cognitions (intentions).

A recent study provides some evidence regarding the role of chronic independent and interdependent self-construal within the TPB, as antecedents to entrepreneurial intentions.

Sampling MBA students from two collectivistic societies, mainland China and Hong Kong, Siu and Lo (2013) found that chronic independence and interdependence were positively associated with entrepreneurial intentions at the individual level. Interdependence was associated with TPB dimensions and moderated the predictive strength of perceived social norms on entrepreneurial intention (Siu and Lo, 2013).

However, although this study contributed to the literature with evidence on direct and moderating effects of self-construal in TPB components and entrepreneurial intensions, its generalizability was limited to one Eastern culture (i.e., Chinese culture). In different countries, persons have a different “mix” of independent and interdependent orientations. It is possible then that cultural orientations, chronic or primed, may lead to different consequences of the TPB components toward entrepreneurial intention. Moreover, different parts of the TPB model may differentially influence entrepreneurial intentions depending on individual-level orientations and interaction with dominant culture-level orientations.

Taken together, the results from research that has examined the TPB components' relationships with entrepreneurial intensions in individualistic and collectivistic cultures confirms expected asymmetries in the role SN have in the two cultural settings. Yet, the relative influence of cultural values on the strength of TPB components over intentions seems to be mixed especially when shifting between country-mean and individual levels of analyses.

The aim of the research was to examine how individual-level self-construal will relate to TPB components and whether independence and interdependence would moderate TPB relationships with entrepreneurial intentions. We aimed to extend the existing models by not only measuring chronic aspects of independent and interdependent self-construal at trait level but also to experimentally manipulate the temporal activation of those and consider relationships with this will mimic results.

The present studies were conducted in Greece, an overall more collectivistic culture than western cultures typically (Hofstede, 2001) yet with an individualist trajectory at family (Georgas, 1989) and person level (Pouliasi and Verkuyten, 2011). Most research on the effects of self-construal on attitudes and cognitive perceptions has been conducted comparing Asian and North American or Northern European individuals who tend to span the extremes of self-construal, By situating the study in this culture it allows to generalize over and above the limited culture samples of previous research.

Given the very limited research examining culture and TPB at person level (mainly one study from one Asian culture) and the mixed findings at culture level analyses we were not in a position to formulate specific hypotheses in the first study which was exploratory in nature. Study 2 aimed to replicate results from the first study in an experimental fashion, and there we formulate hypotheses based on findings from the first study.

## Study 1

Study 1 was designed to investigate relationships between independent and interdependent self-construal and entrepreneurial intentions within the TPB model. It also aimed to examine the moderating effect of chronic independent and interdependent self-construal on the predictive strength of the three components of the TPB (ATT, SN, and PBC) on entrepreneurial intention.

### Participants and procedure

Survey data were collected from 941 Greek university students (353 males, average age 22.27 years, *SD* = 3.08). The majority (42.7%) were engineering students followed by business (12.1%), social science (e.g., psychology, education) (26%), and science (e.g. chemistry, physics, medicine) (19.2%). One hundred and fifty participants (16%) were postgraduate students. Thirty-three percent (33%) of the participants reported that one of their parents owned full time business most of the time while they were growing up, 68% reported that they know an entrepreneur in their close environment.

Surveys were administered individually, through personal contacts. Students were located during leisure activities and asked to voluntarily participate in a research project regarding factors influencing their future career choice. There were no monetary incentives or extra course credits. Data collection took place in the beginning of the 2013 fall semester and lasted for 5 weeks. The survey contained items representing the theoretical constructs along with demographic data. Items referring to the same construct were positioned in different locations throughout the questionnaire. Furthermore, approximately half of the items were negatively worded.

### Measurement of constructs

All constructs were assessed with multi-item self-report scales with known psychometric properties.

The constructs were translated into the Greek language from the English version. The back translation method was used to translate the relevant items (Brislin, 1980) and the few discrepancies between the original English version and the back-translated version resulted in adjustments in the Greek translation. The specific measures used in the analysis, along with sample items of the relevant constructs are outlined below.

#### Self-construal

We assessed independent and interdependent self-construal using the Singelis (1994) self-construal scale (SCS), a measure of chronic or trait self-construal. The SCS is a widely used measure consistent with the theoretical concepts of independence and interdependence identified by Markus and Kitayama (1991). Each of the two dimensions contained 15 items and responses were made on a five point Likert-type scale (1 = strongly disagree, 5 = strongly agree). The independent self-construal subscale (IND) contains items that assess uniqueness in social behavior and related cognitions and emotions (e.g., “I do my own thing, regardless of what others think”). Cronbach's alpha coefficient for this scale was 0.70. The interdependent self-construal subscale (INTER) includes items that asses connectedness in social behavior especially emotions, cognitions, and behavior concerning in-groups (e.g., “It is important to me to respect decisions made by the group”). Cronbach's alpha coefficient for this scale was 0.75.

Previous research using exploratory factor analysis (EFA) provided support for the hypothesis that the SCS comprised two factors (Singelis, 1994). Hardin's et al. (2004) conducted EFA analyses that provided support for the two-factor structure of the SCS. Yet, Hardin's et al. (2004) CFA analyses suggested that three items were found to load on both factors. Moreover, 11 of the 30 SCS items did not load highly on either factor (i.e., neither loading was above 0.3). Our EFA analyses also supported the two factor structure of the 30 items. However, we have found that 14 items had minor cross-loadings on the other factor. CFA of the 30 items loading on one factor resulted in an unacceptable model fit [$\chi_{\left(405,\;N=941\right)}^{2}=2675.82$, *p* < 0.001; RMSEA = 0.118 (90% CI: 0.09–0.13); CFI = 0.540; TLI = 0.506]. CFA results with two corresponding factors, also did not meet acceptable standards of fit [$\chi_{\left(404,\;N=941\right)}^{2}=2856.48$, *p* < 0.001; RMSEA = 0.080 (90% CI: 0.078–0.083); CFI = 0.673; TLI = 0.648]. Although the SCS seems to have a well-defined structure based on EFA analyses, the scale does not provide an acceptable fit when evaluated with CFA (Hardin's et al., 2004; Singelis, 1994). In such cases, the exploratory structural equation modeling (ESEM) approach might be ideally suited (Asparouhov and Muthén, 2009; Morin et al., 2013).

ESEM combines exploratory factor analysis with structural equation modeling (SEM). Like EFA, ESEM also permits the estimation of factor loadings of all items across all factors, so that the problem of fixing the cross-loadings to zero disappears. When the loading matrix of the population includes cross-loadings, ESEM recovers this matrix better than CFA does and it is not subject to its parameter estimation bias. As such, ESEM may be the most appropriate model for determining the factorial structure of the SCS.

Results of the ESEM analyses (conducted with the Mplus, v7 program) indicated that the items used from the SCS and involved two factors had an acceptable fit: [$\chi_{\left(376,\;N=941\right)}^{2}=2118.06$, *p* < 0.001; RMSEA = 0.070 (90% CI: 0.067–0.073); CFI = 0.890; TLI = 0.930].

#### Entrepreneurial intent (INT)

We assessed entrepreneurial intent using Thompson's (2009) originally developed scale. This is a reliable and internationally applicable individual entrepreneurial intent scale. It includes 10 items, four of which are distracter items that act as red herrings and were not included in scale analyses. Sample items are: “Intend to set up a company in the future,” “I have no plans to launch my own business” (reverse scored). Responses to the six items were made on 7-point Likert-type scales (1 = strongly disagree, 7 = strongly agree). Cronbach alpha coefficient for this scale was 0.88. CFA of the 6 items comprising the INT scale resulted in an acceptable model fit supporting the undimensionality of the construct [$\chi_{\left(9,\;N=941\right)}^{2}=118.46$, *p* < 0.001; RMSEA = 0.09 (90% CI: 0.084–0.013); CFI = 0.962; TL = 0.936].

#### Attitudes toward entrepreneurship (ATT)

We assessed ATT using Liñán and Chen's (2009) five items scale. Sample items are: “A career as entrepreneur is attractive for me,” “Among various options, I would rather be an entrepreneur.” Responses to the five items were made on 7-point Likert-type scales (1 = strongly disagree, 7 = strongly agree). Cronbach's alpha coefficient for this scale was 0.90 CFA of the five items comprising the ATT scale resulted in an acceptable model fit supporting the undimensionality of the construct [$\chi_{\left(5,\;N=941\right)}^{2}=15.12$, *p* < 0.001; RMSEA = 0.046 (90% CI: 0.021–0.074); CFI = 0.996; TLI = 0.993].

#### Subjective norm (SN)

We assessed SN using the three items scale from Liñán and Chen (2009). Students were asked: “If you decided to create a firm, would people in your close environment approve of that decision?” Items were: (a) Your close family, (b) Your friends and (c) your fellow students. Responses to the three items were made on 5-point Likert-type scales (1 = total disapproval, 5 = total approval). Cronbach's alpha for this scale was 0.71. CFA provided also a very good fit to the data. CFA of the three items comprising the SN scale resulted in an acceptable model fit [$\chi_{\left(1,\;N=941\right)}^{2}=0.565$, *p* = 0.452; RMSEA = 0.021 (90% CI: 0.017–0.068); CFI = 1.000; TLI = 1.000].

#### Perceived behavioral control (PBC)

We assessed PBC using five items from the scale of Liñán and Chen (2009). Sample items are: “To start a firm and keep it working would be easy for me,” “I can control the creation process of a new firm.” Responses to the five items were made on 7-point Likert-type scales (1 = strongly disagree, 7 = strongly agree). Cronbach's alpha coefficient for this scale was 0.85. CFA also provided a very good fit to the data. CFA of the five items resulted in an acceptable model fit [χ^2^ − (5, *N* = 941) = 62.81, *p* < 0.001; RMSEA = 0.093 (90% CI: 0.087–0.10); CFI = 0.998; TLI = 0.937].

#### Control variables

Student's gender, age, and entrepreneurial role models (having a parent who is an entrepreneur) were used as control variables in the present study. Previous research suggests that demographic characteristics appear to influence perceptions of desirability (Shook et al., 2003). Moreover, having a parent who owns a business is associated with entrepreneurial intent and male students are more likely to have higher entrepreneurial intent compared to females (Crant, 1996).

### Analytic strategy

We tested our data for deviations from normality. We used the AMOS v.7 structural equation modeling program (Arbuckle, 2006) for conducting the analyses with the latent variables. In order to avoid problems associated with common method variance often found in cross sectional survey research, several steps described in the literature (Podsakoff et al., 2003) were taken: approximately half of the items were reverse phrased; items referring to the same latent variable were positioned in different locations in the questionnaire and finally we performed Harman's one-factor test.

We used the two-stage analytic procedure proposed by Anderson and Gerbing (1988) in order to test the structural equation model. First we fitted a measurement model to the data. Next we tested the structural model. During the first step, to test the discriminant validity of the constructs, a measurement model was assessed which allowed the latent variables to correlate freely and constrained each item to load only to the latent variable for which it was a proposed indicator. Next, we examined the change in chi-square (χ^2^), between the measurement model and a model that constrained the correlations among the constructs to be equal. A non-significant χ^2^-value indicates acceptance of the more parsimonious of the nested models. Evidence that common method variance does not account for the observed relationships would be provided if a four factor model, representing each variable as a separate construct, is superior to a one-factor model.

### Testing the moderating effect of self-construals

We have followed the procedure described in the Siu and Lo (2013) paper. Specifically, we used the simple median split approach (Preacher et al., 2005) to generate high and low score groups for the independent self-construal subscale (IND) and the interdependent self-construal subscale (INTER). For IND, cases with scores higher than the median of zero (standardized values) were assigned to the “high score” group and cases below the median were assigned to the “low score” group. The same procedure was followed for the INTER subscale. The number of cases in each group was as follows: High IND = 480; Low IND = 461; High INTER = 487; Low INTER = 454.

According to Siu and Lo (2013) median split is a rather conservative approach that prevents the loss of reliable information. However, this practice of dichotomization has been criticized for the resulting loss of information and reduction in power (MacCallum et al., 2002). Thus, in order to test IND and INTER as moderators, we further adopted a modified version of the Klein and Moosbrugger (2000) approach as implemented in Mplus software. The Klein and Moosbrugger approach automatically handles variable interactions (including latent variables) using the full continuous variable and including an interaction term in the structural equation. That is, one can test latent interaction effects in the structural equation without having to create interactions between individual indicators of the variables. This, mitigates the problem of decreasing reliability of interaction terms, especially when the moderator and/or the independent variable are formed of questionnaire scale items. Related see also Zampetakis et al. (2009) where the Klein and Moosbrugger approach is used for the estimation of a similar interaction effect.

In order to examine whether independent and interdependent self-construal have an effect on the model with the best fit to the data, multi-group analysis of AMOS was then applied. The basic idea was to establish measurement equivalence before comparing predictive paths across groups. First, we tested the invariance of factorial measurement across groups (Byrne, 2001). The measurement model, in which all parameters were freely estimated, was compared to the one in which all factor loadings were constrained to be equal across groups (weak factorial invariance) (Byrne, 2001). Parameters found to be invariant across groups were cumulatively constrained. Then we tested group differences in structural pathways. This procedure provides evidence that group differences in structural pathways are not a function of differences in other parts of the underlying theoretical structure, or instability of the model. For model comparison the CFI can be used. A change in the CFI value less than or equal to −0.01 indicates that we should accept the null hypothesis of invariance (Cheung and Rensvold, 2002).

### Results

#### Descriptive statistics

Table 1 presents means, standard deviations and correlations. In our data, univariate skewness and univariate kurtosis of each indicator variable was less than 0.71 and 1.05 in absolute values, respectively; non-normality was not an issue for our data (West et al., 1995). The mean variance inflation factor (VIF) was 1.36, a value below the suggested cut-off of 4.0, indicating no evidence of severe multicollinearity. Thus, the maximum likelihood estimator was used.

**Table 1:** **Descriptive statistics and inter-correlations for the total sample (Study 1; *N* = 941)**.

	***M***	***SD***	**1**	**2**	**3**	**4**	**5**	**6**	**7**	**8**
Gender^a^	1.62	0.48	–							
Age	22.27	3.08	–0.03	–						
ATT	4.20	1.48	–0.11^**^	0.07^*^	–					
PBC	3.11	1.30	–0.12^**^	0.10^**^	0.47^**^	–				
SN	3.71	0.86	0.00	0.07^**^	0.28^**^	0.26^**^	–			
INT	3.03	1.12	–0.12^**^	0.15^**^	0.61^**^	0.58^**^	0.23^**^	–		
Independent self-construal (IND)	0.00	0.87	0.08^**^	0.12^**^	0.18^**^	0.06	0.27^**^	0.04	–	
Interdependent self-construal (INTER)	0.00	0.89	0.09^**^	0.11^**^	0.14^**^	0.04	0.21^**^	0.07	0.34^**^	–

a Gender is coded: 1 = male, 2 = female;

* p < 0.05 (two tailed);

** *p < 0.01 (two tailed)*.

#### Relationships between individual-level self-construal and TPB dimensions

In order to test the relative importance of independent and interdependent self-construal on the TPB dimensions we regressed ATT, SN, PBC and INT on independence (IND) and interdependence (INTER) using a structural equation model approach. Of the two predictors, independent self-construal was consistently more influential predictor of ATT (standardized coefficients: IND *b* = 0.15, *p* < 0.001; INTER *b* = 0.09 *p* < 0.01, *R*^2^ = 0.04), SN (standardized coefficients: IND *b* = 0.23, *p* < 0.001, INTER *b* = 0.12, *p* < 0.001, *R*^2^ = 0.09), PBC (standardized coefficients: IND *b* = 0.06, *p* = 0.128, INTR *b* = 0.03, *p* = 0.462, *R*^2^ = 0.05), INT (standardized coefficients: IND *b* = 0.02, *p* = 0.596, INTR *b* = 0.07, *p* = 0.07, *R*^2^ = 0.06).

#### CFA and SEM analysis for the whole sample

Results from the CFA suggest an adequate fit to the data: $\chi_{\left(146,941\right)}^{2}=942.93$, *p* = 0.000; RMSEA = 0.076 (90% CI: 0.071–0.080); CFI = 0.920; TLI = 0.917. All factor loadings were significant at the 0.001 level. A model comparison between the unconstrained measurement model and a model that constrained the correlations among the constructs to be equal produced a significant difference in χ^2^, suggesting the presence of discriminant validity among the selected constructs (Δχ^2^ = 296.24, Δ*df* = 4, *p* < 0.001). These results support the multidimensionality and the discriminant validity of the proposed measurement model.

**Figure 1:**
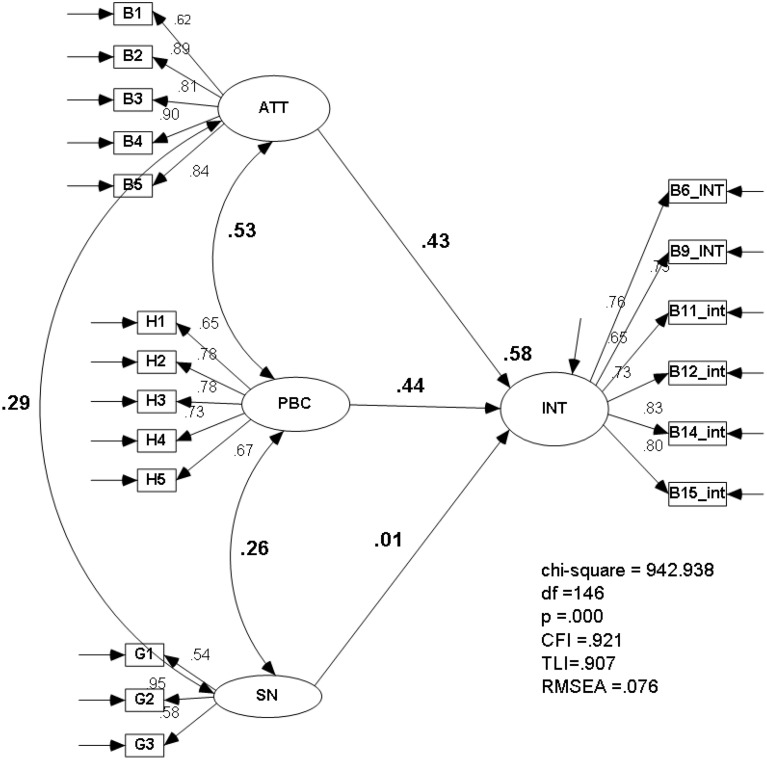
**Structural Model (standardized results)**. Circles represent latent factors; boxes represent indicators. Casual effects are given by arrows connecting circles. Bold numbers over paths are structural weights. Disturbance effects and variance explained for the indicators are omitted for clarity.

The next step in our analysis was to consider the structural model for the whole sample. Results suggest that this model revealed a good fit to the data (see Figure 1). Examining the findings, the structural model for the whole sample model postulated that ATT and PBC have statistically significant direct effects on INT (0.43, *p* < 0.001, two tailed and 0.44, *p* < 0.001, two tailed, respectively).

The direct effect of SN on INT was not statistically significant. Thus, SN was not included in the analyses that follow. No support was found for the effect of control variables on INT.

The proportion of variance (squared multiple correlations) in INT that was explained by the collective set of predictors was 58%. Entering into the structural model, independent self-construal (IND) and the interdependent self-construal (INTER) resulted in a small decrease in model fit (CFI = 0.914), however, the effects of both IND and INTER on INT were not statistically significant. The same pattern of results was found by entering into the structural model only IND or INTER.

#### Multi-group analysis of structural invariance (MASI)

To test the moderating role of self-construals we first conducted a multi-group analysis of structural invariance across groups. First we tested Model 1 (configural invariance) with no equality constrains for both IND and INTER groups. This model revealed an adequate fit to the data for both IND: [$\chi_{\left(292,\;N=941\right)}^{2}=1138.61$, *p* = 0.000; RMSEA = 0.055 (90% CI: 0.052–0.059); CFI = 0.912; TLI = 0.902] and INTER [$\chi_{\left(292,\;N=941\right)}^{2}=1147.22$, *p* = 0.000; RMSEA = 0.070 (90% CI: 0.067–0.076); CFI = 0.915; TLI = 0.900], thus providing evidence of configural invariance across high and low groups of IND and INTER.

For INTER, model 2 (the metric invariance model) displayed an adequate fit to the data, providing evidence of metric equivalence across high and low groups of INTER. All item loadings were found to be invariant across the low and high groups. We used model 2 (the metric invariance model) for testing the structural weights, since it was the model with the better fit (Δχ^2^ = 19.49, Δ*df* = 17, *p* > 0.05). For IND, model 2 also displayed an adequate fit to the data and all item loadings were found to be invariant across the high and low groups.

Model 3 (the structural weights model) displayed an adequate fit to the data for both INTER and IND. For INTER, the structural weights between ATT, PCB and INT were invariant across high and low groups. However, for IND, the structural weight between ATT and INT was not invariant (Δχ^2^ = 7.89, Δ*df* = 3, *p* > 0.05) indicating the condition of partial structural invariance. Our results suggest that interdependent self-construal does not moderate the effect of attitudes toward entrepreneurship (ATT) and perceived behavioral control (PBC) on students' entrepreneurial intentions (INT).

**Figure 2:**
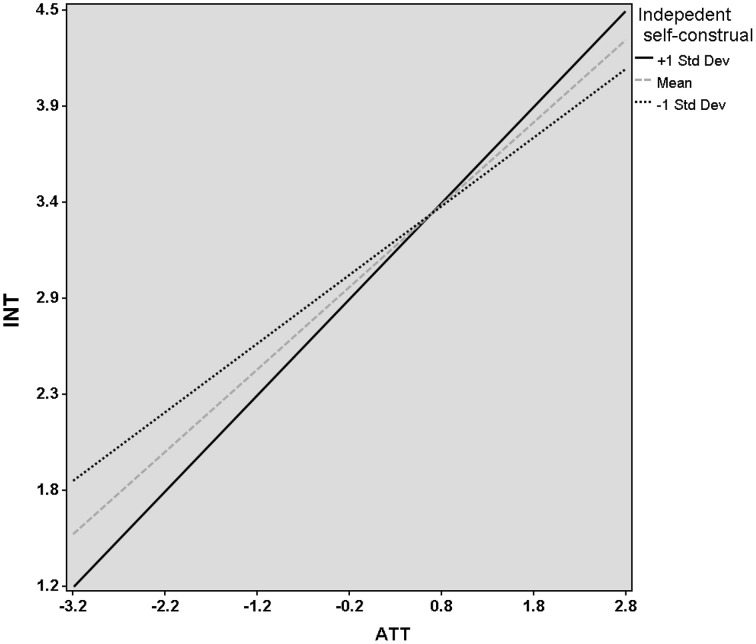
**Relationship between attitudes toward entrepreneurship (ATT) and entrepreneurial intention (INT) for different levels of the moderator variable (independent self-construal)**. The mean, one standard deviation above the mean (+1 SD) and one standard deviation below the mean (−1 SD).

**Figure 3:**
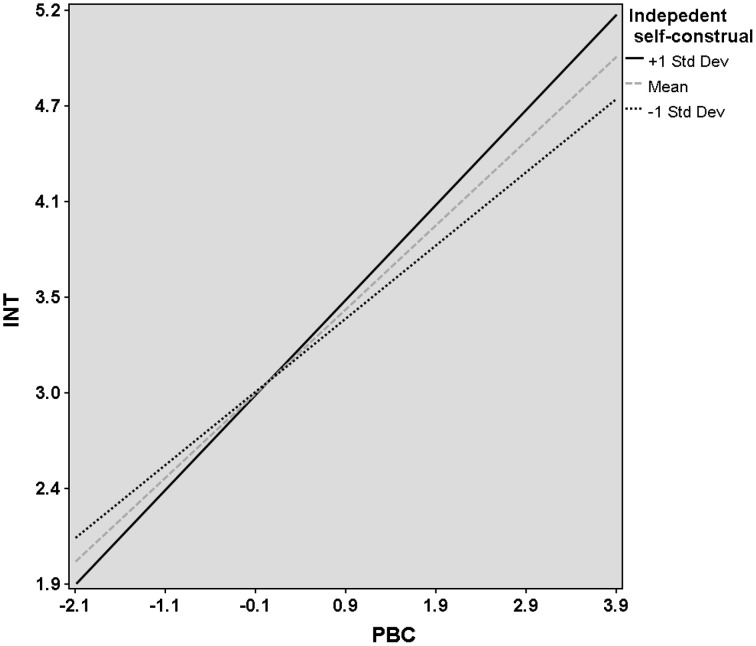
**Relationship between perceived behavioral control (PBC) and entrepreneurial intention (INT) for different levels of the moderator variable (independent self-construal)**. The mean, one standard deviation above the mean (+1 SD) and one standard deviation below the mean (−1 SD).

We found that the relationship between ATT and INT was significant stronger when the independent self-construal was high (*r* = 0.48, *p* < 0.001) compared to low (*r* = 0.38, *p* < 0.001). That is to say, we have found a significant positive interaction effect between ATT and independent self-construal (*r* = 0.08, *p* < 0.001; see Figure 2). Moreover, we have found a significant positive interaction effect between PBC and independent self-construal (see Figure 3). However, if both interaction effects were entered in the model, the interaction of PCB by independent self-construal was rendered non-significant.

We have found the same pattern of results with the application of the Klein and Moosbrugger (2000) approach implemented with Mplus. Specifically, for the IND dimension, the structural equation model that included the IND × ATT interaction term (AIC = 36989.40; BIC = 37197.82) yielded a better fit to the data than did the model which excluded it (AIC = 36998.07; BIC = 37201.64). The value of the interaction standardized coefficient was significant [β = 0.10 (*p* < 0.001)]. On the other hand the structural equation model that included the INTER × ATT interaction term (AIC = 37051.24; BIC = 37259.66) yielded a worse fit to the data than did the model which excluded it.

In summary, this first study examined the role of person-level self-construal in the full TPB model. We found that ATT and PBC predicted students' entrepreneurial intention. In line with others studies (Krueger et al., 2000; Liñán and Chen, 2009), we have found that the effect of SN on entrepreneurial intentions was not statistically significant. The findings of this first study, guided the hypotheses and design of the second study which was experimental. Specifically, we wanted to replicate and extend the findings from the first study, by manipulating self-construal with an experimental approach.

## Study 2

Study 2 aimed to replicate findings from study 1 using an experimental design. In this study we manipulated the temporal availability of independent and interdependent self-construal to examine the impact of both on entrepreneurial attitudes and intentions. Social contingent models such as the “culture as situated cognition” framework (Oyserman and Lee, 2008) see cultural mind-set as dependent on salient social contextual aspects that are typical for a given culture and give rise to individualistic or collectivistic mind-sets (Oyserman and Lee, 2008; Oyserman et al., 2009). In that sense individuals' chronic cultural orientation tendencies are the trait-level equivalent of temporarily activated cultural orientations, and these are examined in the second study. Based on results from the first study we expected that temporary activation of independent self-construal will result in higher entrepreneurial attitudes and intentions and that temporary activation of independence would moderate relationships between the entrepreneurial attitudes and intentions.

### Participants

Participants were 79 undergraduate students (48 males, 31 females) from three Greek state universities in southern Greece. Students were enrolled in introductory courses in business administration and management. Most of the participants were engineering students (55.1%), with other majors including social sciences (28%), humanities (10%), and sciences (6.9%). All participants received course credit for completing their questionnaires. Participant ages ranged from 17 to 22 years, with mean age of 20.55 (*SD* = 1.05) years. Participants were randomly assigned to one of the two experimental conditions (independent or interdependent self-construal prime) and were run by the authors in non-interacting groups of 5–7 members in each group.

### Procedure

On arrival at the laboratory/class, participants were informed that the study examined the factors influencing students' entrepreneurial intent and that anonymity was guaranteed. After providing informed consent, participants were provided a three-page booklet to fill out. On the first page and prior to the priming manipulation, participants completed: (1) a measure of mood [five positive (Alert, inspired, determined, attentive, active) and five negative feeling states (Upset, hostile, ashamed, nervous, afraid)], where responses were made on a five point Likert-type scale (1 = never, 5 = always), (2) the probability of starting their own business sometime in the future (on a scale from 0 to 100) and (3) demographic information (age, gender).

In the second page the priming procedure was modeled after the “Sumerian warrior” story procedure (Trafimow et al., 1991, study 2; see Kafetsios and Hess, 2015). The “Sumerian warrior” prime emphasizes distinguishing the self from others by acting on self-interest (independent) versus emphasizing interconnections by acting on social/family interest (interdependent). Participants were asked to read a paragraph about a central character who was engaged either in a competitive one-man sport or a team sport, both resulting in success and then rated the central character in the story on a number of characteristics using seven point scales (1 = not at all, 7 = very much see Appendix). The adjectives within the paragraph and those in the following evaluation were selected from related tasks such as the scrambled sentence task (Oyserman and Lee, 2008).

### Measurement of dependent variables

In the third page of the booklet, all participants answered questions about the study's main dependent variables: attitudes toward entrepreneurship (ATT) and entrepreneurial intent (INT). ATT and INT were assessed using the same measures as in Study 1, and responses were made on a 10 point Likert-type scale (1 = absolutely disagree, 10 = absolutely agree). Cronbach's alpha coefficient for ATT was 0.88 and 0.87 for the independent and interdependent self-priming conditions, respectively. For INT, Cronbach's reliability was 0.89 and 0.91 for the two groups respectively.

As a manipulation check, participants also completed three items from the Singelis (1994) independent self-construal scale (to verify that participants in the independent self-priming condition score higher than participants in the interdependent self-priming condition). The items used were: “*I enjoy being unique and different from others in many respects,” “I do my own thing, regardless of what others think,” “I feel it is important for me to act as an independent person.”* Responses were made on a 10 point Likert-type scale (1 = absolutely disagree, 10 = absolutely agree). The three items were averaged to create an independence index. Cronbach's alpha coefficient for the three items was 0.76 and 0.78 for the independent and interdependent conditions respectively. Due to the small sample size, we used bootstrapping procedures (resampled 1000 times and used the percentile method to create 95% Confidence Intervals (CI).

### Results

Prior to priming, the two groups did not differ significantly in their levels of positive and negative affect, [*F*_(1, 78)_ = 0.318, *p* = 0.574] and [*F*_(1, 78)_ = 0.636, *p* < 0.428], respectively. The priming procedure was successful, as indicated by the results of the manipulation check [*F*_(1, 78)_ = 42.524, *p* < 0.001]: participants in the independent self-priming condition scored higher in the independence index (*M* = 7.35, *SD* = 1.67; 95% CI: 6.80–0.58; CI: 6.81–7.89) than participants in the interdependent self-priming condition (*M* = 5.20, *SD* = 1.25; 95% CI: 4.84–5.55). This difference was accompanied by a large effect size (Cohen's δ = 1.46). These results provide evidence that the priming task resulted in a shift toward the independent and the interdependent self, regardless of any chronic differences in self-construal.

The effect of self-construal manipulation on attitudes toward entrepreneurship (ATT) was significant [*F*_(1, 75)_ = 5.463, *p* = 0.022], after controlling for students' gender and entrepreneurial role models (β = 0.51, *p* = 0.022). As expected, participants primed with an independent self-construal perceived entrepreneurship to be more attractive (*M* = 6.74, *SD* = 2.24; 95% CI: 6.05–7.44) than did those primed with an interdependent self-construal (*M* = 5.81, *SD* = 1.89; 95% CI: 5.24–6.31). This difference is accompanied by a medium effect size (Cohen's δ = 0.44).

However, the effect of the manipulation on students' entrepreneurial intent (INT) after controlling for students' gender and entrepreneurial role models, was marginally non-significant [*F*_(1, 75)_ = 3.445, *p* = 0.067]. Moreover, the interaction effect of self-construal manipulation and ATT on INT was non-significant, *b* = 0.06, 95% CI: [(−0.312) − 0.432].

In line with results from Study1 and prior research (Ybarra and Trafimow, 1988) we found that attitudes toward entrepreneurship were more strongly associated with intentions for students primed with independence (*r* = 0.556) (vs. interdependence; *r* = 0.518). Yet, this difference in correlations was small and not statistically significant (*z* = 0.049, *p* = 0.96; see (Weaver and Wuensch, 2013) for the procedures used for comparing correlations).

## General discussion

The present set of studies examined within-culture differences in independent and interdependent self-construal as antecedents to entrepreneurial intentions within the context of the Theory of Planned Behavior (TPB, Ajzen, 1991). We found that in Greece, both chronic (Study 1) and temporarily activated (Study 2) independent self-construal were associated with more positive attitudes toward entrepreneurship. Additionally, we found evidence that independent self-construal moderated the relationship between attitudes and intentions such that positive attitudes were associated with entrepreneurial intentions among people who strongly (vs. weakly) construe the self as independent. The main strength of the present studies is that they identify the conditions and implications of key cultural dimensions of independence and interdependence at individual level for judgment (Aaker and Maheswaran, 1997; Shavitt et al., 2006) and entrepreneurial preference (Bird, 1988; Busenitz and Lau, 1996) within a single culture.

Although entrepreneurial intent has been examined from a cross-cultural perspective (e.g., Busenitz and Lau, 1996; Tiessen, 1997; Mitchell et al., 2000; Liñán and Chen, 2009), to our knowledge this is one of the first studies to examine within-culture individual-level cultural orientations as antecedents to entrepreneurial intentions and attitudes in some depth. In that respect the studies and results reported herein extend a body of research on the cognitive model of entrepreneurial intention within TPB (Krueger et al., 2000; Siu and Lo, 2013). The results from both studies suggest that for Greek students who place more value on personal autonomy, individual initiative, self-sufficiency and a relative detachment from the situational and relational context, independent self-construal can positively affect their attitudes toward entrepreneurship and as shown in study 1, those attitudes can have a significant influence on their entrepreneurial intentions.

These findings are in keeping with cultural contingency approaches to cognitive models of entrepreneurship (Busenitz and Lau, 1996; Siu and Lo, 2013). Attitudes are multi-component constructs comprising by affective and cognitive components (Drolet and Aaker, 2002). The affective component concerns the encoding of emotions and feelings associated with the attitudinal object, and the cognitive component is conceived as containing the encoding of attributes and beliefs about the object which can be affected differently by independent and interdependent self-construal. Given that an independent self-construal is also associated with higher positive affect at the individual (Van Hemert et al., 2007) and interpersonal (Kafetsios and Nezlek, 2012) levels, it is plausible that the affective component of interdependent orientation may be driving those effects. Therefore, a formidable task for future research would be to explore the possible role positive and negative affect can have on the said connections.

In the introduction the conceptual analysis of the large part of the culture and entrepreneurship cognition literature distinguished between culture and individual level of analyses. At the cultural level, the results from the present studies are in line with previous research in countries that reported higher individualist values (i.e., United States; Krueger et al., 2000; Peterman and Kennedy, 2003). It is also interesting that, in the first study, chronic interdependence was not influential on entrepreneurial intentions as reported in research in collectivistic cultures (Liñán and Chen, 2009; Siu and Lo, 2013) and also that subjective norms were positively associated with independent self-construal. From the perspective of the TPB, our results support previous research findings that perceived behavioral control, that is, people's perceptions of their ability to perform a given behavior, is a robust predictor of entrepreneurial intentions (Kolvereid, 1996; Krueger et al., 2000; Peterman and Kennedy, 2003). Furthermore, SN was found to be a non-significant predictor of intentions, a finding consistent with the results from studies in other countries (Autio et al., 2001). It is thus possible that the making of entrepreneurial career decisions in Greece is of such importance that young people are not likely to be influenced by the opinions of others.

Theoretically, the results from the second study are in line with social contingency models of self-understanding such as the “culture as situated cognition” framework (Oyserman and Lee, 2008): when the private independent self was made more accessible, people perceived entrepreneurship more favorably. In line with applications in educational settings (e.g., Oyserman et al., 2002b), the results suggest that examining culturally situated mind-set antecedents to attitudes toward entrepreneurship may be a fruitful research avenue for applied research on the topic. Although the priming procedure of study 2 was successful results, do not confirm our expectation that the temporary activation of independence would moderate the relationship between entrepreneurial attitudes and intention. It is plausible that the effects resulting from the temporarily activated self-construal fades out fairly quickly over time compared to chronic self-construal, so that the expected interaction is difficult to manifest in detectable effects (Gardner et al., 1999). Clearly more research is needed to test this.

Results reported herein, are also indicative of the mix of collectivistic and individualistic tendencies that characterize cultural orientation at culture and individual levels in Greece. At culture level Greece is a more collectivistic country (Hofstede, 2001). Therefore, one explanation for the discrepancies observed between our research in Greece and other collectivistic countries (i.e., China), may be suggestive of differences at culture level between collectivism in Greece and other collectivistic countries like China. Yet, another explanation is also possible. As the results from the present studies shed further light by specifically footing self-construal orientations at the individual level, these differences in changes in societal and cultural norms may also mean that individualism is gaining ground (Georgas, 1989; Pouliasi and Verkuyten, 2011). Treating independent and interdependent self-construal as separate at person level may allow future research for more predictive power by comparing people who are high in both, low in both and unmitigated in self-focus or other-focus (Konrath et al., 2009).

Our research has further implications and applications. Given the association of attitudes and perceived behavioral control with intentions, in our sample, education programs could pay attention to positively influencing students' attitudes toward entrepreneurial activity and increasing perceived behavioral control for creating a new firm. Furthermore, although the role of mass media and especially television has been acknowledged in stimulating entrepreneurship, still very little is known about the influence of the media on occupational choices or on the attitudes toward entrepreneurship. Our results may provide a starting point toward this direction by indicating the cultural frame that messages aiming at increasing attitudes toward entrepreneurship should have. According to the “culture as situated cognition” perspective (Oyserman, 2011), message effectiveness increases when chronic cultural frame, entrepreneurship message tailoring, and momentarily salient cultural frame all match. Our results suggest that messages placing an emphasis on individualistic cultural frames are suitable for increasing attitudes toward entrepreneurship. In the same vein, in order educators to encourage young students to engage in entrepreneurial behavior, they should not highlight connections with family or friends.

The two studies reported here are not without limitations. First, in Study 1 we used only single source data. Therefore, the interpretations of correlation evidence should be interpreted with caution. Second, we knowingly targeted younger participants who are likely to have a more pronounced independent self-construal. Future research should strive to include older age entrepreneur start-ups. Third, we only assessed the role of individual differences for a single culture with its own unique mix of individualism and collectivism. Future research should examine the role of individual differences in self-construal in, for example, more individualist cultures. Fourth, in the present research we used the Singelis (1994) self-construal scale. However, other authors have used other scales to assess chronic self-construal. For example Siu and Lo (2013) used a new measure of self-construal developed by Lu and Gilmour (2007). Furthermore, from the 42 items of the original self-construal scale Siu and Lo (2013) used only 13 items (four items for the independent self-construal and nine items for the interdependent self-construal) with adequate psychometric properties, raising validity issues. Future research could benefit from the application a common framework for the assessment of self-construal. Fifth, in the present study, we used the exploratory structural equation modeling (ESEM) approach, which is a recent advancement in latent variable modeling. However, the measurement model fit indexes suggest that there is still room for improving the measurement of self-construal. Finally, future research could also benefit how the cognitive model of entrepreneurial intention is affected by other cultural orientations, such or power distance or uncertainty avoidance.

## Conclusion

There is a growing consensus that entrepreneurial behavior is embedded within social and cultural norms and values; thus, a greater understanding of the relationship between cultural issues and entrepreneurial activity is necessary given entrepreneurship's implication for national and regional development and growth. In the present paper, we examine whether and how the ways that people utilize to perceive themselves to be linked (or not) with other people (interdependent and independent self-construal) influence entrepreneurial cognition models. Our results, convincingly suggest that independent self-construal moderates the effect of attitudes toward entrepreneurship on entrepreneurial intent providing evidence that cultural change may have an impact on individual attitudes toward entrepreneurship.

### Conflict of interest statement

The authors declare that the research was conducted in the absence of any commercial or financial relationships that could be construed as a potential conflict of interest.

## Footnotes

^1^This paradoxical finding can be understood in terms of support perceptions in more collectivistic environments, see Kafetsios and Nezlek (2012).

## Appendix

We would like you to read the following paragraph. After reading the paragraph, we would like you to evaluate the extent to which the following sentences reflect the main character's characteristics.

### Independence priming

Nikos is a unique and competitive person. He is training in Tae Kwon Do persistently for a long time. He has participated in many competitions and won a few of them. He is a dynamic person and competes with decisiveness in order to reach his goals. He prefers to be independent and devotes his free time to training than entertainments. In his last game, he felt all these years' efforts were justified. Generally, Nikos believes that if one really wants he can achieve whatever he wants in his life.

Nikos is: optimistic, enthusiastic, energetic, happy, disciplined, independent, dynamic, hard, decisive, alone, competitive, calm.

### Interdependence priming

Nikos is a member of the local football team. He trains daily with his fellow players for many hours. After all this time with them, their relationship is strong and have understanding. Due to the spirit of camaraderie, the cohesion and the support the team has managed to reach the first division. Nikos believes in his team because he believes in the abilities of his fellow players and the cooperation with them. Reaching this year's cup final feels with happiness and pride that they all tried hard for the team's success.

Nikos: shows trust in others, shows respect for others, shows understanding for others, is devoted to others, is proud of others, shows gratitude to others, despises others, is cooperative with others, is jealous of others, shows support to others, is hard with others, has “filotimo” (honor).
